# Genomic signatures of selection, local adaptation and production type characterisation of East Adriatic sheep breeds

**DOI:** 10.1186/s40104-023-00936-y

**Published:** 2023-11-06

**Authors:** Boris Lukic, Ino Curik, Ivana Drzaic, Vlatko Galić, Mario Shihabi, Luboš Vostry, Vlatka Cubric-Curik

**Affiliations:** 1 Faculty of Agrobiotechnical Sciences Osijek, J.J, Strossmayer University of Osijek, Vladimira Preloga 1, 31000 Osijek, Croatia; 2 Department of Animal Science, Faculty of Agriculture, University of Zagreb, Svetošimunska Cesta 25, 10000 Zagreb, Croatia; 3 Department of Maize Breeding and Genetics, Agricultural Institute Osijek, Južno predgrađe 17, 31000 Osijek, Croatia; 4 Czech University of Life Sciences Prague, Kamýcká 129, 165 00 Praque, Czech Republic

**Keywords:** Composite-likelihood ratio, East Adriatic sheep, Extreme ROH islands, Genomic selection signatures, Integrated haplotype score, Number of segregating sites by length

## Abstract

**Background:**

The importance of sheep breeding in the Mediterranean part of the eastern Adriatic has a long tradition since its arrival during the Neolithic migrations. Sheep production system is extensive and generally carried out in traditional systems without intensive systematic breeding programmes for high uniform trait production (carcass, wool and milk yield). Therefore, eight indigenous Croatian sheep breeds from eastern Adriatic treated here as metapopulation (EAS), are generally considered as multipurpose breeds (milk, meat and wool), not specialised for a particular type of production, but known for their robustness and resistance to certain environmental conditions. Our objective was to identify genomic regions and genes that exhibit patterns of positive selection signatures, decipher their biological and productive functionality, and provide a "genomic" characterization of EAS adaptation and determine its production type.

**Results:**

We identified positive selection signatures in EAS using several methods based on reduced local variation, linkage disequilibrium and site frequency spectrum (eROHi, iHS, nSL and CLR). Our analyses identified numerous genomic regions and genes (e.g., desmosomal cadherin and desmoglein gene families) associated with environmental adaptation and economically important traits. Most candidate genes were related to meat/production and health/immune response traits, while some of the candidate genes discovered were important for domestication and evolutionary processes (e.g., *HOXa* gene family and *FSIP2*). These results were also confirmed by GO and QTL enrichment analysis.

**Conclusions:**

Our results contribute to a better understanding of the unique adaptive genetic architecture of EAS and define its productive type, ultimately providing a new opportunity for future breeding programmes. At the same time, the numerous genes identified will improve our understanding of ruminant (sheep) robustness and resistance in the harsh and specific Mediterranean environment.

**Supplementary Information:**

The online version contains supplementary material available at 10.1186/s40104-023-00936-y.

## Background

Around 12,000 BP, sheep (*Ovis aries*) were among the first animals domesticated by humans during the Neolithic Revolution [1–3]. Along with goats, sheep were historically selected and preferred for their milk, meat, and wool production. They thrive outdoors and adapt well to local conditions, which in most cases are not suitable or sufficient for cattle. Sheep production is deeply rooted in the eastern Adriatic and was introduced during the Neolithic migrations [4–6] so all populations have the same non-native origin. Croatian production primarily includes traditional systems without specialized breeding for carcass or milk yield [7] making local breeds such as Istrian sheep, Cres Island sheep, Pag Island sheep, Krk Island sheep, Rab Island sheep, Lika Pramenka sheep, Dalmatian Pramenka sheep, and Dubrovnik Ruda sheep valuable genetic resources. These breeds are bred for milk, meat and to a limited extent wool, but are hardy and resilient in harsh environments. They are informally (also from the breeder’s point of view) considered well suited for meat and dairy production, although they are not favored over international breeds in any of these production directions.

With the development of molecular genetics, the identification of selection signatures reflecting natural or artificial selection has become possible, and numerous methods have been developed [8–11]. Four methods developed to identify selection signatures in livestock populations, all based on within-population approaches: i) extremely high SNP incidence in ROH ("extreme ROH islands" or "eROHi"), ii) integrated haplotype score (iHS), iii) number of segregating sites by length (nSL), and iv) composite likelihood ratio test (CLR), are widely used to identify hard and soft selection signals [12–18]. For example, many previous studies on Mediterranean sheep used statistical methods with medium density genomic data and found candidate genes for coat color, morphology, milk, and wool [19–24].

An analysis by Ciani et al. [25] identified Croatian breeds from the eastern Adriatic as part of the distinct "Balkan sheep group" with considerable genetic variation, which was confirmed by a broader diversity study using Infinium HD SNP arrays [7]. All of these breeds are distributed over an area approximately 1,000 km long and 50 km wide (more information provided in the Additional file 1). Although each breed or population is kept in a small but specific area, such as islands or peninsulas, they still form a unique group with the same origin, breeding environment, and socio-agricultural background. Understanding their genomic variation is critical for sustainable breeding to address environmental and economic challenges, while evidence of positive selection patterns offers insights into domestication, evolution, and post-Neolithic change in Europe.

Using a comprehensive approach involving four selection signature methods, we aimed to uncover adaptive selection signals, profile production types, and elucidate gene functions within selection patterns. These analyses are novel for autosomes in EAS as a member of the "Balkan sheep group" breeds/populations where selection signatures have not been identified previously. We have located selection signature regions, identified candidate genes, and characterized their biological/molecular functions. In general, the knowledge gained in this study improves our understanding of the genetic background of production capacity in EAS, which will help to improve EAS breeding.

## Methods

### Data and genotyping

The animals included in this study were selected and collected in collaboration with the National Gene Bank of the Croatian Ministry of Agriculture, and all procedures with the animals were performed in accordance with national and European ethical protocols and guidelines. The animals were raised by registered breeders at different locations in Croatia, and information was available on their origin and the exact location of the farm. Sampling of closely related animals (parents with offspring, full or half siblings) was avoided.

A total of 196 animals of East Adriatic sheep breeds [7], were analysed as metapopulation (EAS) in this study, which includes Istrian sheep (*n* = 25; from 8 flocks), Cres Island sheep (*n* = 19; from 5 flocks), Pag Island sheep (*n* = 45; from 16 flocks), Krk Island sheep (*n* = 19; from 3 flocks), Rab Island sheep (*n* = 18; from 17 flocks), Lika Pramenka sheep (*n* = 18; from 18 flocks), Dalmatian Pramenka sheep (*n* = 25; from 18 flocks), and Dubrovnik Ruda sheep (*n* = 26; from 17 flocks). Short descriptions of the breeds and pictures can be found in the Additional file 1. While collecting samples, our aim was to collect samples from high number of flocks in order to obtain representative sample and to avoid close genetic relationships, therefore we collected samples from overall 102 flocks. With the exception of the Pag Island sheep, sample sizes were similar. More detailed information on the samples (sex, map locations and coordinates etc.) in this study can be found in Additional file 2. As described in the Background, we decided to analyse EAS as one metapopulation in this study. Our preliminary analysis was performed for each of the breed separately, however, the signals of selection were less clear and more power was obvious in the case of metapopulation due to the higher sample size. Furthermore, we conducted an analysis of the decay of linkage disequilibrium (LD), which we also performed (Additional file 3). This analysis revealed a consistent LD decay pattern across all breeds, providing additional support for the effectiveness of employing a large metapopulation strategy. All animals were genotyped using the Ovine Infinium® HD SNP BeadChip 600 K (606,006 SNPs). Skin tissue samples from the ear were collected as part of the regular sampling of local autochthonous breeds by the National Gene Bank and from the blood, from which DNA was isolated using a commercial kit (DNeasy Blood and Tissue Kits, Qiagene, Germany). Plink 1.9 software [26] was used for quality control and data management. Of the genotypes obtained, we analysed only autosomal SNPs whose chromosomal position was assigned. SNP positions were based on the sheep genome assembly ARS-UI_Ramb_v2.0, and duplicate or misassigned SNPs were excluded from the analysis. SNPs missing more than 5% of genotypes and SNPs with an Illumina GenCall score < 0.7 were excluded from analysis. Sheep missing > 10% of the genotypes were also excluded from further analysis, while the thresholds to filter out all SNPs with a minor allele frequency of < 1% or due to deviations from Hardy–Weinberg equilibrium (HWE) were < 0.01%. Finally, to avoid duplicate and related individuals (first- and second- degree relatives), the degree of common ancestry was calculated for each pair of individuals using a threshold of identity by descent (IBD) > 0.18. After performing these quality control criteria, the final data set consisted of 500,831 SNPs and 195 sheep (98 females and 97 males).

### eROHi analysis

ROH analysis was performed with the detectRUNS package in R software [27] and SNP & Variation Suite (v7.6.8 Win64; Golden Helix, Bozeman, MT, USA, www.goldenhelix.com) using the consecutive runs method, a "windowless" method that searches the genome for ROHs regardless of window size. The criteria for detecting runs were determined by recommendations for HD SNP data [28]. Thus, the minimum ROH length was set to 1 Mb, the minimum number of consecutive homozygous SNPs in the called ROH was set to 15, the minimum SNP density for the ROH was set to at least one SNP every 0.1 Mb, the maximum distance (gap) between consecutive homozygous SNPs was set to 1 Mb. In longer runs, one heterozygous SNP was allowed due to the possibility of genotyping or assignment errors. Finally, detected ROHs were categorised based on their length in Mb as follows: 1–2 Mb, 2–4 Mb, 4–8 Mb, 8–16 Mb, and > 16 Mb. To establish a significance threshold, the frequency of ROHs was calculated and normalised by their chromosome means, with the transformed value represented as −log_10_ (*P*) from the right tail of the normal distribution for each of the chromosomes separately. In this way, overrepresentation of extreme ROH islands due to chromosome size was avoided. SNPs with −log_10_ (*P* value) ≥ 4 were considered outliers, whereas only regions with consecutive outliers were considered as selection signals [29].

### iHS and nSL analyses

For the analyses of iHS and nSL, the required haplotypes were phased from SNP data in SHAPEIT2 software [30] with 200 conditioning states and a window size of 2 Mb. The iHS statistic, based on the concept of Extended Haplotype Homozygosity (EHH) [31], was calculated in R software using the rehh package [32]. Two-tailed *P* values for iHS [33] were calculated as −log_10_(2Φ(−|iHS|)), where Φ(*x*) represents the Gaussian cumulative distribution function. Sliding windows of 0.5 Mb with an overlap of 0.01 Mb with adjacent windows were used, and windows with three or more SNPs exceeding the threshold (−log_10_ (*P* value) ≥ 4) were considered as selection signatures. Since recombination rates are highly heterogeneous across the genome, we also used the nSL method, which is similar to the iHS method but was proved to be more robust in detecting selection signatures, regardless of the variation in recombination rate [34]. The nSL statistics were calculated with the selscan program [35] using the default parameters. Thus, a gap scale parameter of 0.02 Mb and a maximum allowable distance between two SNPs of 0.2 Mb were used, whereas all SNPs below 0.05 were removed. The results of this analysis were then normalized to 100 frequency bins and to two-tailed *P* values for each SNP across all chromosomes using the program norm [35]. Using the same approach as the iHS method, selection signatures were determined based on the normalized values.

### CLR analysis

The SweeD software, Linux version 3.0 [17], was used to calculate composite likelihood ratios (CLR) to test local reductions in nucleotide diversity along chromosomes. The reimplementation of the SweepFinder CLR test [8] in SweeD provides a robust representation of the genetic hitchhiking process caused by the chromosomal linkage of advantageous alleles to neighbouring polymorphisms that represent signatures of strong directional selection [36]. The SweeD CLR score represents the likelihood of a selective sweep in a tested region based on a denominator (likelihood for a null hypothesis empirically derived across all SNP positions assuming no selection) and a numerator (likelihood of a sweep in a tested region). The grid size determining the number of regions to be tested was set at 10,000 per chromosome. Larger grid sizes were also tested (up to 100,000 per chromosome) without significant impact on the final results. The top 0.1% of hits were interpreted as loci under selection, resulting in a genome-wide threshold of 10.92.

### Gene annotation and functional gene analysis

Functional biological interpretation of the discovered selection signatures was performed by conducting GO analyses [37]. Only significant selection signatures (regions whose initial and final positions overlapped) identified by at least two different methods were analysed (Additional file 4). Annotation of candidate genes was performed with the Ensembl [38] tool BioMart using the latest available assembly (ARS-UI_Ramb_v2.0). A total of 19 regions with an average region length of 0.970 Mb met our criteria and were subjected to further analysis (https://www.ensembl.org/biomart/martview). Searching for the associated genes using BioMart yielded 4,984 gene transcripts or 349 genes with unique Gene Stable IDs. The identified genes were further classified and subjected to GO and protein domain (INTERPRO domains) enrichment analysis using publicly available software DAVID [39, 40]. Gene IDs were analysed using the online tool the Database for Annotation, Visualization and Integrated Discovery (DAVID), resulting in 25 functional similarity clusters. The clustering provides useful approach to group a large number of genes which are functionally similar, and therefore make gene annotations more understandable in the context of biology. The clustering algorithm uses Kappa statistics to measure how much genes are shared between two annotations. It also employs fuzzy clustering to categorize similar annotation groups based on these Kappa values. Essentially, if annotations have a greater number of common genes, they are more likely to be grouped together.

### Production and breeding type characterization

We also characterised the production mode of EAS by analysing the properties of positive selection signals ("candidate genes") using the QTL (Quantitative Trait Loci) database for sheep (https://www.animalgenome.org/cgi-bin/QTLdb/OA/index). The QTL database contains sheep QTL/association data from 201 previously published studies and more than 3,000 QTLs. In these analyses, we focused on candidate genes whose significance overlapped with all methods used in this study. QTLs from the QTL database were annotated using the GALLO R package [41] to query the Animal QTLdb (https://www.animalgenome.org/cgi-bin/QTLdb/index) for previously identified QTLs in regions of interest.

## Results

### Detected signatures of selection

In this study, we used four different intrapopulation methods for highly dispersed genomic information with an average spacing of 5 kb between SNPs, which provided us with a powerful tool to accurately detect positive genomic selection signatures at high resolution. A total of 165 genomic regions with positive selection signals were identified, of which 19 were identified by at least two different methods. Therefore, we conservatively focus our discussion and interpretation only on putative genes located in genomic regions of high confidence. These genes are consequently treated as candidate genes specific to the investigated East Adriatic sheep breeds.

**Fig. 1:**
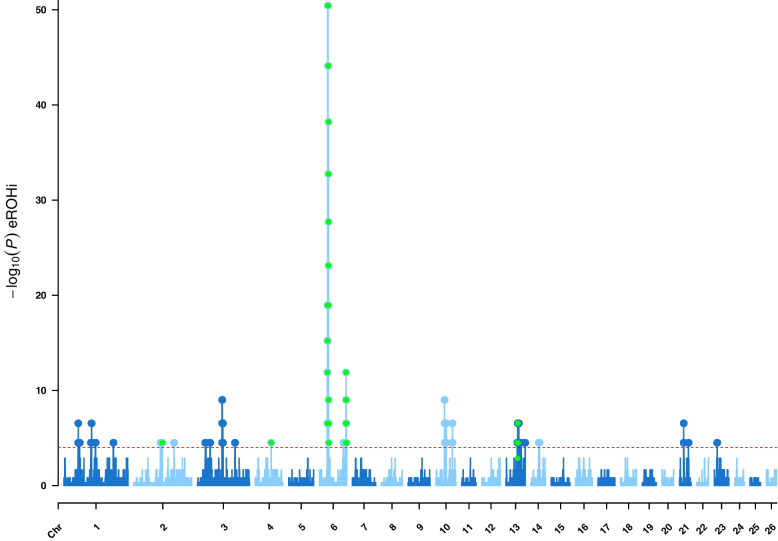
Manhattan plot of genome-wide eROHi analyses on East Adriatic sheep breeds from Croatia. Horizontal red dashed line marks the significance threshold of –log_10_ (*P* value) = 4. Chromosomal regions from 100 kb up and down the genome were highlighted as selection signatures for all detected eROHi-s. Regions confirmed by at least one of the other methods were highlighted by green colour

**Fig. 2:**
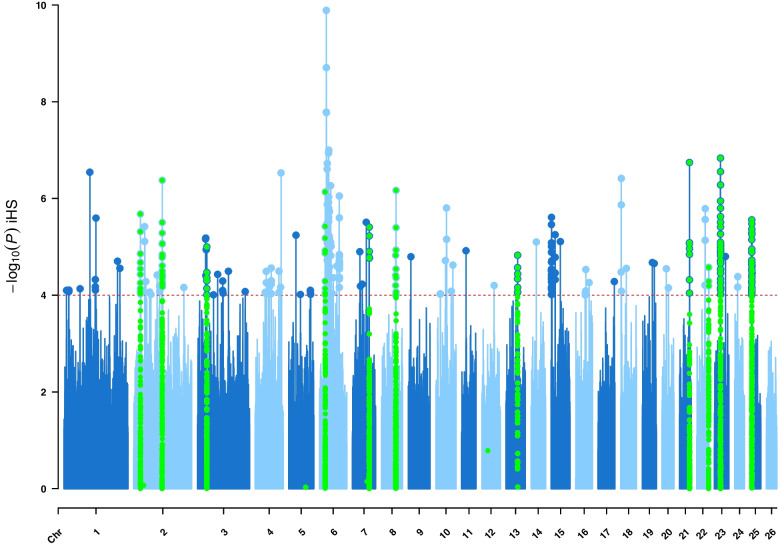
Manhattan plot of genome-wide iHS analyses on East Adriatic sheep breeds from Croatia. Horizontal red dashed line marks the significance threshold of –log_10_ (*P* value) = 4, and if the windows with three or more SNPs exceeded this threshold, they were considered as selection signatures. Regions confirmed by at least one of the other methods were highlighted by green colour

**Fig. 3:**
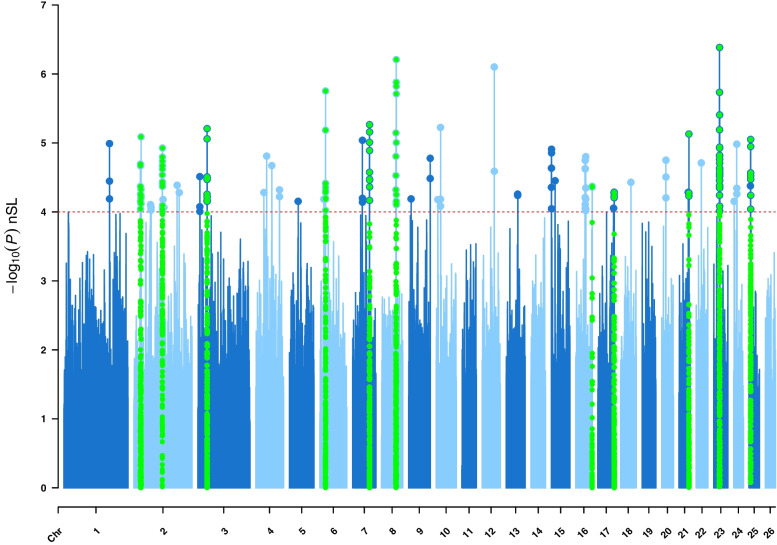
Manhattan plot of genome-wide nSL analyses on East Adriatic sheep breeds from Croatia. Horizontal red dashed line marks the significance threshold of –log_10_ (*P* value) = 4, and if the windows with three or more SNPs exceeded this threshold, they were considered as selection signatures. Regions confirmed by at least one of the other methods were highlighted by green colour

**Fig. 4:**
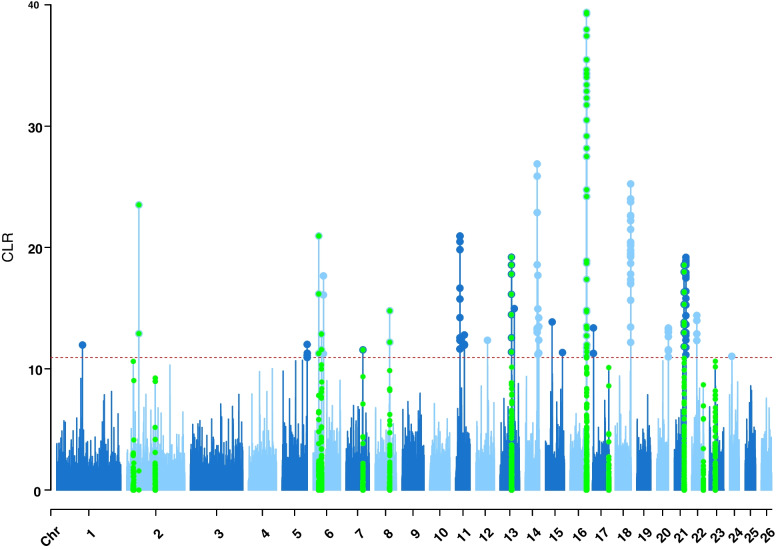
Manhattan plot of genome-wide CLR test on East Adriatic sheep breeds from Croatia. Horizontal red dashed line marks the significant genome wide threshold of 10.92, based on the top 0.1% hits, which were interpreted as selection signatures. Regions confirmed by at least one of the other methods were highlighted by green colour

Genomic regions detected as selection signatures by four different methods are shown as Manhattan plots in Fig. 1, 2, 3 and 4 and listed in Table 1. The threshold for detection of selection signals by the eROHi method was set to 4 [−log_10_ (*P* value)], and chromosomal regions from 100 kb up and down the genome were highlighted as selection signatures for all detected eROHi-s. In this way, 48 genomic regions were detected as selection signatures only by the eROHi method, while six of them were also confirmed by at least one of the other methods used and were finally highlighted in the Manhattan plots by green colour and vertical lines. More detailed information on the genomic regions detected and the overlap between the methods can be found in Additional file 5.

**Table 1:** Candidate regions and genes detected using all methods

Region	Chr	Position, bp^a^	Length, Mb	Methods	Genes
1	2	27,846,647–29,059,562	1.213	CLR-IHS-NSL	*WNK2, SUSD3, ECM2, ASPN, OMD, OGN, NOL8, W5PEV5*
2	2	30,200,000–31,190,000	0.990	NSL-CLR	*
3	2	121,400,000–123,491,237	2.091	ALL	*ZSWIM2, FAM171B, ZC3H15, W5Q575, FSIP2*
4	3	39,840,000–40,930,000	1.090	NSL-IHS	*ETAA1*
5	4	68,150,000–69,110,000	0.960	IHS-ROHS	*TAX1BP1, EVX1, W5PII1, HOXA10, HOXA9, W5PJ00, HOXA6, HOXA4, HOXA3, HOXA2, W5PJE3*
6	6	24,035,512–25,350,000	1.314	CLR-IHS-NSL	*EMCN, C6H4orf54*
7	6	32,870,000–40,660,000	7.790	IHS-ROHS-CLR	*CCSER1, TIGD2, FAM13A, PIGY, MED28, FAM184B, LCORL, W5P3G4, KCNIP4*
8	6	112,846,266–116,882,460	4.036	CLR-ROHS	*W5PDR8, SORCS2, W5PQN1, AFAP1, ABLIM2, W5PUD5, GRK4, MFSD10, SH3BP2, FAM193A, RNF4, W5Q0Q8, POLN, W5Q1Y2, TACC3, W5Q3F5, LOC101104663, LOC101105412, GAK, W5Q513, W5Q5J9*
9	7	71,934,333–73,092,556	1.158	CLR-IHS-NSL	*W5QJ23, SGPP1*
10	8	60,900,000–61,880,000	0.980	NSL-IHS	*MTFR2, MAP7*
11	8	62,319,974–63,470,000	1.150	CLR-IHS-NSL	*LOC101113683, W5NRH7, NHSL1*
12	13	484,42,121–50,598,845	2.157	CLR-IHS-ROHS	*RNF24*
13	13	50,563,961–51,826,354	1.262	CLR-ROHS	*ADAM33*
14	16	68,890,399–71,643,038	2.753	NSL-CLR	*W5P6Z2, CEP72, BRD9, W5Q3L2, W5Q3M4, W5Q3S1, W5Q4G2*
15	17	69,010,000–70,000,000	0.990	NSL-CLR	*W5P400, SMTN, RNF185, PIK3IP1, SFI1, PRR14L, DEPDC5*
16	21	41,901,704–42,960,000	1.058	CLR-IHS-NSL	*GPR137, CCDC88B, RPS6KA4, SLC22A11, NRXN2, MAP4K2, W5PK24, MEN1, CDC42BPG, NAALADL1, ZFPL1, TMEM262, FAU, MRPL49, SYVN1, SLC22A20P*
17	22	49,880,000–50,810,000	0.930	IHS-CLR	*W5Q425, CFAP46, W5Q8X7, KNDC1, W5QB54, CALY*
18	23	25,350,000–26,730,000	1.380	CLR-IHS-NSL	*RNF138, RNF125, DSG2, DSG3, DSG4, DSG1, DSC1, DSC2, LOC101116442*
19	25	6,540,000–8,070,000	1.530	NSL-IHS	*W5NZP4, IRF2BP2, W5NZU5, RBM34*

^a^Start and end position of the genomic region

^*^ No candidate genes found

The strongest signals were detected in two regions on *Ovis aries* chromosome 6 (OAR6), the region between 35.613 and 37.361 kb, where 155 SNPs exceeded the −log_10_ (*P* value) of 50, followed by OAR10 and OAR3. The majority of ROH segments detected in the sheep genome were short regions with a length of 1–2 Mb.

Genomic regions identified as selection signatures by iHS and nSL methods are shown as Manhattan plots in Fig. 2 and 3. To avoid selecting only SNPs above certain thresholds, we defined a window of 500 kb containing three or more SNPs with −log_10_ (*P* values) greater than 4. In this way, 22 genomic regions showing a positive selection pattern were detected by the iHS method, while 15 of them also overlapped with the other methods used (highlighted in green).

Regions exceeding the above conditions for the iHS method were located on OAR2, OAR3, OAR4, OAR6, OAR7, OAR8, OAR13, OAR15, OAR21, OAR22, OAR23 and OAR25. The four strongest signals detected by the iHS method were located at OAR6, OAR23, OAR21 and OAR2, as highlighted in Fig. 2. The average length of the identified genomic regions was 1.1 Mb.

The nSL method identified 17 regions that had a positive selection signature pattern, while 13 were confirmed by other methods. The genomic regions identified by the nSL method were located on OAR2, OAR3, OAR6, OAR7, OAR8, OAR13, OAR15, OAR16, OAR17, OAR20, OAR21, OAR23, OAR24 and OAR25. Although these two methods showed high correlation [9] in detecting selection signatures, surprisingly no complete (or at least high) overlaps were found. Of the total 28 selection signatures detected by iHS and nSL, they overlapped only in nine genomic regions, confirming that the difference in recombination rate affects the results of our analyses. The three strongest signals detected by the nSL method were located at OAR23, OAR8, and OAR6, as highlighted in Fig. 3, while the average length of the detected regions was 1 Mb.

Genomic regions identified as selection signatures by the method CLR are shown as a Manhattan plot in Fig. 4. In ﻿﻿this study, the method CLR with a defined threshold of 0.1% highest hits identified 78 selection signatures, 21 of which overlapped with other methods. The genomic regions identified by the method CLR, which were also confirmed by other methods, were on OAR2, OAR6, OAR7, OAR8, OAR13, OAR16, OAR17, OAR21, OAR22 and OAR23. The three highest CLR values were found in genomic regions on OAR16, followed by OAR2 and OAR6, which also overlapped with other methods. The average length of the regions found was 0.95 Mb, which is comparable to the average length of the other regions.

### GO analyses

GO and enrichment analysis results are presented only for genes found in the overlapping genomic regions by two or more methods because this approach increases the reliability of the identified genes (otherwise, too many genes would have been shown but their interpretation would have been less reliable [39, 40]). The genes that were annotated in 19 overlapping genomic regions in our analyses are listed in Table 1. Of the total 4,984 gene transcripts, 342 genes (Additional file 6) were identified with unique Gene Stable IDs and subjected to GO analysis using the software DAVID. Functional classification analysis of genes based on the functional similarity algorithm [40] identified 10 gene groups (Additional file 7), providing a clearer picture of the extensive gene list from this study.

This analysis allows us to categorize a large number of genes based on their biological background, which facilitates the interpretation of their functions. These 10 groups consisted of 128 functionally related genes (see Table 1) that were assigned according to their chromosomal position and are considered candidate genes in this study. Modified Fisher's exact *P* value (EASE score), which ranks the biological significance of the gene groups, was above 3.00 and was highest for the first three groups, 3.43 for the first group and 3.39 and 3.37 for the second and third groups, respectively. Functional annotation analysis identified 116 annotation terms and their associated genes in this study (Additional file 8). Finally, functional annotation cluster analysis identified 25 annotation clusters corresponding to biological functions, which are discussed below.

The terms from the first five annotated clusters (Additional file 4) exceeded a significance of *P* ≤ 0.05 but only the first two exceeded Benjamini–Hochberg correction for multiple hypothesis testing. In the first cluster, the most abundant and enriched GO terms and INTERPRO domains (IPR009122, GO:0030057, IPR009123 and IPR000233) were associated with desmosomal cadherin, desmoglein, and desmocollin, with the following associated genes *DSG3, DSC1, DSG4, DSC2*, *DSG2* and *DSG1* on OAR23. All three protein families and their isoforms are dense adhesion complexes required for tissues to withstand mechanical stresses and are responsible for maintaining tissue integrity and facilitating cell–cell communication [42, 43]. As described above, they are mainly related to immune response and properties of tissue, which is very important in the context of local adaptation in sheep. In the second cluster, the major enriched GO terms and INTERPRO domains (IPR020479, IPR009057, IPR001827, IPR001356 and IPR017970) were linked to homeobox genes (*HOXA10*, *HOXA3*, *HOXA2*, *HOXA4*, *HOXA6* and *HOXA9*). These are a highly conserved group of genes found not only in animals but also in plants and fungi. They belong to the class of transcription factors that play a key role in developmental processes [44]. As previously shown, several morphological and developmental traits in sheep (e.g., number of vertebrae, inner thigh, tail) were found to be associated with this group of genes, and therefore this analysis confirms their importance in the genetic structure of East Adriatic sheep breeds. The enriched GO terms and INTERPRO domains from the third, fourth, and fifth clusters had significant *P* values but did not exceed the threshold for multiple comparisons (third cluster—GO:0005509, IPR002048 and IPR011992, all associated with calcium ion binding and hand domain genes; fourth cluster—GO:0061630 and IPR001841: all associated with zinc finger and ubiquitin protein ligase; and fifth cluster—GO:0004842 and IPR000408: all associated with protein transferase activity and regulation of gene expression).

### Production type characterisation

**Fig. 5:**
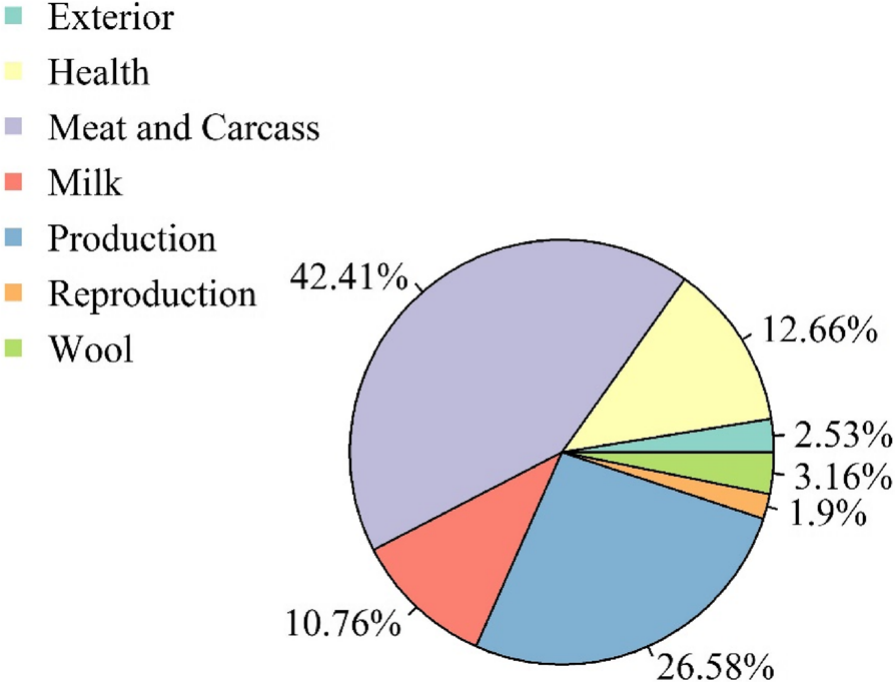
Genes classified based on the Sheep Animal QTL database. QTLs were annotated using the GALLO R package and the Animal QTLdb for the previously identified QTLs in regions of interest. The identified genes are classified based on their associations with traits from the database

The results of the QTL database analyses are shown in Fig. 5, and Additional file 9 and 10. In Fig. 5, the identified genes are classified based on their previously found associations with traits from the database. Additional file 9 and 10 show the significantly enriched traits/types per chromosome and genome, respectively, as determined by QTL enrichment analysis. The area of the bubbles represents the number of QTLs observed for that class per chromosome, while the colour represents the FDR-adjusted *P* value as −log_10_ (*P* value) (the darker the colour, the smaller the *P* value). In addition, the *x*-axis shows the richness factor for each QTL, which is the ratio between the number of observed and expected QTLs.

Because we have identified numerous and very specific genomic regions in this study using four different methods, we have carefully described the biological functions of the genes in these regions. However, for the breeding context, the genomic background of these candidate genes is usually less clear because of complex mechanisms such as pleiotropy, epistasis or hitchhiking effects [41]. Figure 5 shows the percentage of each QTL type in the identified candidate regions in a pie chart, indicating that EAS breeds are formed primarily for meat and carcass traits, followed by production, health traits, and finally milk.

## Discussion

### Production type characterisation

A careful review of the old literature and knowledge [45] on sheep breeding in the Croatian Mediterranean region focuses on adaptation to the Mediterranean environment, but without a systematic breeding approach. Historically, this was an area without a sufficient food base for livestock (unlike the eastern and continental areas of Croatia) and with a low standard of living, so the main products were meat, milk, and wool. At the beginning of the nineteenth century, more than 1,000,000 sheep were counted in the eastern Adriatic region of Croatia, which was the highest per capita number of sheep in Europe. All these facts (large sheep population, low standard of living) and local trends in sheep breeding led sheep farmers to focus mainly on their own needs. Therefore, focusing on meat/production, health and milk was a logical breeding decision. In addition, commercial wool production required more specialized breeds with industrial support (e.g., Merino), which was not the case in this breeding area. Since meat production was the main breeding objective, the sheep also had to be well adapted to the harsh environment, so health traits were a very logical breeding objective that was also complemented by natural selection. This is a fairly realistic explanation for our results related to economically important traits or production type characterization for EAS. The overall list of candidate genes identified in this study also supports our conclusion, as most of them are associated with meat/production traits in several species and with health and disease resistance. In addition, the enriched GO terms and INTERPRO domains from the first two gene clusters could be considered the most important in our study, especially since they are consistent with the number of identified individual candidate genes and with the first three trait groups based on the annotated QTLs (meat/production and health traits).

### Candidate genes related with meat and carcass traits

On OAR2, *SUSD3* (sushi domain containing 3) is a gene found to be associated with intramuscular fat content in pigs [46]. Another two very interesting genes have been discovered on OAR2, the *ASPN* and the *OGN* genes, which were precisely mapped and described [47]. Both genes belong to the leucine-rich proteoglycan (*SLRP*) family and play important roles in various functions such as collagen fibrillogenesis, cell growth, cell differentiation, and migration [48]. This was confirmed by a proteomics and gene expression approach [49], in which thin-tailed sheep had higher *ASPN* expression levels compared with thick-tailed sheep and was also validated as a protein within the collagen fibril organizing group. In the aforementioned QTL study in pigs [47], *ASPN* and *OGN* genes were identified to be significantly associated with carcass traits at a genome-wide level, specifically loin and neck meat weight, shoulder meat weight, and daily gain (110–210 d). On OAR3, gene *FAM171B* is associated with meat colour in cattle [50], but its function is still unclear. On OAR4, we identified the gene *EVX2*, which is associated with the limbs and genital buds in sheep [51] and with mammary gland and spinal cord development in pigs [52], confirming its possible function in sex organ development.

The phosphatidylinositol glycan anchor biosynthesis class Y or *PIGY* gene is a member of the PIG gene family and has an important function in cell–cell interactions. In the study about the copy number variations surrounding this gene in sheep [53], it was found to be associated with growth traits and the type traits of chest girth and cannon bone circumference, suggesting that this gene could be used as a marker for breeding purposes. It has also been associated with meat and carcass quality [54]. Mediator complex subunit 28 (*MED28*) is a gene with function in regulating smooth muscle cell differentiation and maintaining stem cell population. This gene is also associated with carcass characteristics and bone weight [55]. Two studies found that the *SORCS2* gene is involved in intracellular sorting and transport of various neurotrophic factors, transmembrane receptors, and synaptic proteins associated with a variety of cellular processes, including neuronal function, differentiation, and synaptic plasticity in different species [56, 57]. Decreased *SORCS2* expression increased oxidative stress and resulted in an enhanced oxidative stress response in primary neurons [57]. The *SORCS2* gene was also found to control lipid metabolism in cattle [58]. It is showed that the *ABLIM2* gene is required for normal neuronal function in humans, rats, and mice [59], whereas in dogs and pigs [60] it encodes an actin-binding muscle protein. Important *GRK4* gene was found in OAR6, near which two ROH islands (ROH100 and ROH101) were found that are unique to Blackhead Persian and Nguni sheep and responsible for chemokine pathways [61]. Another research [62], found a role for the *GRK4* as well as the *MFSD10* gene in lipid storage, fat cell regulation, and fat tail deposition in Mediterranean sheep. The *SMTN* gene or smoothelin is expressed in various tissues such as smooth muscle, adipose tissue, cardiac muscle, and skeletal muscle [63], with two main isoforms. It has a function in controlling muscle contraction [64], but no association has been found with economically important traits in livestock.

Kinase non-catalytic C-lobe domain containing 1 or *KNDC1*, plays a key role in many signal transduction pathways that contribute to protein recognition and functional regulation. In chickens, it has been associated with mammillary layer thickness and mammillary density [65], whereas in pigs it has been associated with the fatty acid profile in IMF along with the *CALY* gene from the same region [66]. In the gene expression study in sheep, *KNDC1* was shown to be involved in the regulation of protein phosphorylation and was upregulated in fast-red muscle compared with slow-red muscle [67].

### Candidate genes related with growth production traits

The *ECM2* gene, which is involved in the extracellular matrix, has been associated with growth in humans [68]. Within the same cluster on OAR2, another member of the same *SLRP* family, the *OMD* gene (osteomodulin or osteoadherin), was discovered to be involved in body development processes, such as regulation of the diameter and shape of collagen fibrils and bone formation [69]. The gene *ZC3H15* has been associated with skull shape in mice [70]. Another gene on OAR6, *LCORL* (ligand-dependent nuclear receptor corepressor), is associated with various body size traits in sheep, humans, horses, and cattle [71–82]. As for the *POLN* gene, it has been reported to have an effect on adult body size in some sheep in the United States [83]. The function of the *TACC3* gene on OAR6 has not been fully elucidated, but it is generally suspected that it may be involved in the processes of cell growth and differentiation. Expression of this gene is upregulated in some cancer cell lines and at embryonic d 15 in mice [84]. In OAR16, only one region (2.753 Mb long) was confirmed by two methods (nSL and CLR). In this region two genes, *CEP72* and *BRD9*, play important roles in bovine feed efficiency [85].

### Candidate genes related with health traits

The *WNK2* gene on OAR2 is associated with tumour suppressive function in humans [86], and when this gene is knocked down, it leads to accelerated tumour growth in mouse models. Another gene on OAR2, *ZSWIM2* was associated with perinatal mortality in cattle [87]. At OAR3, we identified an interesting gene, *ETAA1* or Ewing tumour-associated antigen 1, that is differentially expressed in fat and short-tailed sheep [88], whereas in humans it is associated with body fat distribution [89]. In cattle, this gene is a strong indicator of mastitis resistance and somatic cell score [90]. Next, on OAR6, the *SH3BP2* gene plays an important role in natural killer cell-mediated cytotoxicity, which is likely responsible for the natural immunity/resistance of local sheep breeds [61]. Cyclin G-associated kinase (*CAK*) is a protein-coding gene on OAR6. Diseases associated with *GAK* include prostate sealing ring cell adenocarcinoma and Parkinson's disease 15, autosomal recessive early onset. Nance-Horan syndrome-like 1 protein (*NHSL1*), located on the OAR8 is related to metabolism and has been associated with subclinical ketosis and resistance to gastrointestinal nematodes in adult sheep [91] and tick parasites [92], which is important for better environmental adaptation. The following 4 genes were located on OAR17. The ubiquitin-protein ligase or *RNF185* gene has a function in the endoplasmic reticulum (ER) associated degradation pathway (*ERAD*) and is associated with mitochondrial autophagy processes. The *PIK3IP1* gene is involved in the negative regulation of phosphatidylinositol 3-kinase activity and in the negative regulation of phosphatidylinositol 3-kinase signal transduction. These two genes are associated with longevity [93] and disease resistance [94, 95] in cattle, as shown by GWAS.

The *SFI1* gene, or centrin-binding protein also on OAR17, has been associated with reproductive traits [96], metabolic digestion [97], and interestingly, cold adaptation, as it affects basal metabolic rate [98]. In addition, in humans, this gene was proved to be associated with glycosylated haemoglobin [99], a marker of average blood glucose levels. The gene Proline Rich 14 Like or *PRR14L* in this region is mainly studied in humans in the context of blood metabolism (increase in monocytes and decrease in neutrophils) and leukaemia gene expression profiles [100], whereas in sheep was proved to be differentially expressed between certain breeds [101].

On OAR21, the *CCDC88B* gene encodes a coiled-coil domain-containing protein 88b, a poorly annotated gene specifically expressed in spleen, bone marrow, lymph nodes, and thymus. CCDC88B protein is also abundantly expressed in immune cells, including CD4^+^ and CD8^+^ T lymphocytes, and in myeloid cells. Loss of CCDC88B protein expression has pleiotropic effects on T lymphocyte functions, including impaired maturation in vivo, markedly reduced activation, decreased cell division, and impaired cytokine production (IFN-γ and TNF) in response to T cell receptor activation during the course of *Plasmodium berghei* infection in vivo [102]. Another interesting study [103] was conducted to explore the resistance of different sheep breeds to gastrointestinal nematodes. Analysing the transcriptome of abomasal lymph node tissue, they found a total of 25 significant (*P* < 0.05) gene interaction networks. The gene interaction network with the largest number of focal molecules (*n* = 18) including the *NAALADL1* gene on OAR21 was assigned to infectious disease, cell-to-cell signalling and interaction, and cell movement. Also on OAR21, *FAU* or FAU Ubiquitin Like and Ribosomal Protein S30 Fusion is a protein coding gene. In the transcriptome analysis of adipose tissue from two fat-tailed sheep breeds [104], was found the specific gene expression patterns in adipose tissue with 47 common highly expressed genes, of which 28 genes affected *FAU* gene. Diseases associated with *FAU* included sarcoma and osteogenic sarcoma [104]. Related pathways included viral mRNA translation and the integrated breast cancer pathway. On the OAR23, important gene family was identified containing the ring finger genes *RNF138* and *RNF135*, both of which belong to the ubiquitin-binding protein family and have strong activities [105, 106]. They play a special role in the regulation of T cells and the immune response. *RNF135* was found to be associated with biological pathways that affect human body size [68].

### Candidate genes related with milk production

An interesting gene on OAR6 is the endomucin or *EMCN* gene, which has been associated with growth and carcass traits in sheep [107], with meat marbling in cattle [108], but also with milk fat yield in sheep [109], which is the first described selection signature related to milk production in this study. *EMCN* is a membrane-bound glycoprotein expressed on the surface of the endothelium in venules and capillaries [110], and its main function is inflammation regulation, so it may be of particular interest in the context of environmental adaptation and disease resistance.

For the *FAM13A* (family with sequence similarity member 13A) gene located in the second important region on OAR6, a detailed GWAS study found an association with lung disease in humans [111], whereas other studies in sheep confirmed an association with milk yield [112] and body/bone weight in cattle [55, 113]. In the same region on OAR6, *MED28* was found to be associated with milk production traits in sheep [112] and expressed in the mammary gland during lactation [114]. The former study also confirmed the association of the *FAM184B* gene (member B of the family with sequence similarity 184) with somatic cell score and milk production traits. *FAM184B* is known to be expressed in adipose and skeletal muscle tissue and during skeletal development [55, 113]. *DEPDC5* or *DEP* domain containing 5 is a gene located on OAR17 and involved in stimulus response and associated with growth traits [115]. Whole-genome analyses [116] have also shown that this gene family plays a role in the regulation of lactation in sheep. On OAR22, *CFAP46* or cilia and flagella associated protein 46, has been associated to lactose content [117] whereas *RBM34* or RNA-binding protein 34 gene on OAR25 has role in nucleic acid binding and nucleotide binding, and it was found by GWAS study on cattle to be associated with milk yield [118].

### Candidate genes related with wool traits

The *AFAP1* (actin filament associated protein 1) gene on OAR6 plays a role in some characteristics of yearling wool of one-year-old Chinese sheep with fine wool, such as the cleanliness of the fleece [119]. On OAR6, two gene families, desmoglein or *DSG* protein with four members, *DSG1*, *DSG2*, *DSG3* and *DSG4*, and desmocollin protein or *DSC* with two members, *DSC1* and *DSC2*, were identified in this study. These genes are involved in immune response in sheep [91], but more interestingly, they have been shown to affect fibre properties [120, 121] and hair growth and follicle structure [122]. For this reason, this gene may be particularly important for adaptation to local environmental and climatic conditions and for characterizing production type. On OAR25, *IRF2BP2* gene is very interesting and quite important for wool characteristics. It is the Interferon regulatory factor 2 binding protein 2 which acts as a transcriptional coregulator and it was found to be involved in the fleece variation between the hairy/long coat and short/woolly fleece phenotypes [123]. Also, it was confirmed in another study about the functionality of these genes in relation to immune and reproduction response, where high and low gene signalling variants resulted in different hair phenotypes [124].

### Candidate genes related to reproduction traits

The coiled-coil serine rich protein 1 or *CCSER1* gene on OAR6, which encodes a proline-rich protein, plays an important role in mitosis and cell division and has been linked to several human cancers [125]. SNPs in the intronic region of this gene have been associated with male fertility traits in sheep, such as ejaculate quality [126], and together with the *TIGD2* gene with growth and carcass traits in cattle [127], body weight in salmon [128], and meat quality traits in ducks [129]. The *TIGD2* gene has also been associated with resistance to disease and bacterial infection in cattle [85], making it an important candidate in our study. The *KCNIP* gene (potassium voltage-gated channel interacting protein 4) also located on OAR6 has an important function in regulating transmembrane transport of potassium ions. Several GWAS studies confirmed the association of this gene with male fertility [126] and growth traits [55, 130, 131]. On the second important region on OAR6, the *RNF4* gene was found to play an important role in litter size in pigs, being involved in a number of reproductive physiological processes [132]. Only one region on OAR7 was detected as a positive selection signal. The *SGPP1* gene plays an important role in ovine fertility traits, where increased expression in the endometrium of the non-gravid uterine horn was detected. *SGPP1* expression also increased in the placenta late in gestation [84, 133] and in lipid metabolism [134].

### Candidate genes related to environmental adaptation

The gene on OAR2, *NOL8*, which has been associated with circulating fasting glucose levels in mice [135] and may be important for energy expenditure in harsh environments. It has also been identified as a candidate gene for hunting ability in dogs [136] and carcass traits in cattle [137]. The gene *FSIP2* also on OAR2, was found to be associated with fecundity in sheep [138]. In the context of domestication, the *FSIP2* gene is very interesting because it was previously identified as a gene important for domestication [138, 139], i.e., a gene associated with adaptation rather than production traits. Moreover, the haplotypes of this gene strongly resemble those of Asian mouflon and other wild sheep relatives (snow sheep and argali) but not those of domestic sheep. The *HOXa* gene family, consisting of six genes (*HOXA2*, *HOXA3*, *HOXA4*, *HOXA6*, *HOXA9* and *HOXA10*) and possibly important for EAS adaptation, are located on OAR4. The *HOXA* gene cluster or homeobox A is a group of conserved genes throughout the animal kingdom that encode several transcription factors responsible for nervous system, body, and spinal development [140, 141]. This gene cluster is particularly important in an evolutionary context because a single mutation in this cluster results in drastic body shapes [140, 142]. Several studies have found associations of this region with similar traits related to developmental functions and morphological traits, such as the number of thoracic vertebrae [143] and fat tail development [144] in sheep, inner thigh development [145] in cattle, and body structure traits [146] in pigs. This region has been identified in numerous selection signature studies based on various methods in different breeds of sheep [147–150].

The *FAM193A* gene on OAR6 was identified as a candidate for adaptation in Moroccan sheep [151]. Two regions were identified on OAR13, each containing only one gene. The gene from the first region (*RNF24*) is a gene responsible for visual function in sheep and has also been found as a selection signature by other authors [138, 152], and the resulting changes are most likely related to domestication. Vision plays a critical role in animal survival, and many studies have shown that visual acuity is weaker in domestic animals (e.g., chickens, dogs and ducks) compared with their wild ancestors [153–155]. Thus, the functional role of this selection signal with respect to domestication remains to be explored. The second region on OAR13 localized the gene *ADAM33*, which belongs to the disintegrin and metalloprotease domain family. It is another gene with a possible function in environmental adaptation, as it plays a role in several biological processes, including muscle development and neurogenesis, whereas in humans it is mainly associated with the immune response and allergic asthma [156–158].

In a study on cattle [159], it was found that the *NRXN2* gene (located on OAR21 in sheep), among several other genes, is associated with adaptation, particularly climate adaptation, such as adaptation to tropical humidity and harsh environments. An interesting study [160] was conducted to investigate the key genes and pathways involved in the response to pain in goats and sheep by transcriptome sequencing. The analysis was performed on the dorsal root ganglion (DRG*),* which is involved in the transmission of pain to the central nervous system and exhibits various pathophysiological changes in chronic pain. Transcriptome analysis in sheep revealed the higher activity of the gene *CDC42BPG*, which regulates the activity of small GTPases (which act as molecular switches or timers in many basic cellular processes such as signal transduction, protein biosynthesis, translocation of proteins across membranes, etc.). Also on OAR21, the gene encoding Hmg-CoA reductase 1 or *SYVN1* degradation may mediate resistance to diabetic retinopathy, as shown in the study on mice [161]. *SYVN1* is an important member of the E3 ligase complex in the *ERAD* pathway that removes misfolded and non-functional proteins from the ER, keeps the ER stable, and reduces ER stress. *SYVN1* also inhibits apoptosis induced by ER stress [162, 163].

## Conclusions

In this study, we identified selection signatures of East Adriatic sheep breeds using several methods, including reduced local variation, linkage disequilibrium and frequency spectrum (eROHi, iHS, nSL, and CLR). Analysis of selection signatures identified numerous and specific candidate genomic regions and genes (e.g., desmosomal cadherin and desmoglein gene family, and *HOXa* gene family) that may be important not only for economically important traits but also for adaptation to specific production and environmental conditions. The majority of candidate genes were related to meat/production and health/immune response traits, which seems to be a realistic historical reflection of breeding practices in the Croatian Adriatic region. This was also confirmed by GO and QTL enrichment analysis. Our results will contribute to a better understanding of the breeding potential of EAS, its unique adaptive genetic architecture and its relationships with other populations, and eventually provide a new opportunity to exploit its genomic background in future sustainable breeding programs. Even though these procedures (incorporating knowledge about selection signatures and population structure) aren't simple and readily executable, we hold the view that incorporating this information in breeding programs should become a mandatory aspect in addressing worldwide shifts and striving for enhanced sustainable production, placing an emphasis on improved adaptation to diverse environmental conditions.

### Supplementary Information

**Additional file 1: Description S1.** Contains short description of the breeds and representative pictures of animals used in this study.

**Additional file 2: Table S1.** Data Info. Contains basic information (breed and number of samples) about the animals used in this study.

**Additional file 3: Fig. S1.** LD decay. Contains results from the LD decay analysis.

**Additional file 4: Table S2.** Gene clusters. Contains results from the functional annotation cluster analysis using DAVID software, with identified annotation clusters corresponding to biological functions.

**Additional file 5: Table S3.** Regions overlap. Contains information on detected selection signatures and regions whose positions overlapped by at least two different methods.

**Additional file 6: Table S4.** Identified genes ID. Contains detailed information (Gene ID, chr, start and end positions in bp) about the genes identified within genomic regions detected as selection signatures.

**Additional file 7: Table S5.** Functional gene groups. Contains results from the functional classification analysis of genes based on the functional similarity algorithm using DAVID software.

**Additional file 8: Table S6.** Functional terms. Contains results from the functional annotation analysis with annotation terms and their associated genes using DAVID software.

**Additional file 9: Fig. S2.** Significantly enriched traits per chromosome, as determined by QTL enrichment analysis. The area of the bubbles represents the number of QTLs observed for that class per chromosome, while the colour represents the FDR-adjusted *P* value as –log_10_ (*P* value) (the darker the colour, the smaller the *P* value). The *x*-axis shows the richness factor for each QTL, which is the ratio between the number of observed and expected QTLs.

**Additional file 10: Fig. S3.** Significantly enriched traits per genome, as determined by QTL enrichment analysis. The area of the bubbles represents the number of QTLs observed for that class per genome, while the colour represents the FDR-adjusted *P* value as –log_10_ (*P* value) (the darker the colour, the smaller the *P* value). The *x*-axis shows the richness factor for each QTL, which is the ratio between the number of observed and expected QTLs.

## Data Availability

Genotypic data representing eight Croatian sheep breeds are deposited and publicly available at https://doi.org/10.5061/dryad.pg4f4qrsn.

## Abbreviations

CLR: Composite-likelihood ratio test
EAS: East Adriatic sheep breeds from Croatia
EHH: Extended Haplotype Homozygosity
eROHi: Extreme ROH “islands”
GO: Gene Ontology
iHS: Integrated haplotype score
nSL: Number of Segregating sites by length
QTL: Quantitative Trait Loci
ROH: Runs of homozygosity
