# Supplementary material for: Genomic signatures of selection, local adaptation and production type characterisation of East Adriatic sheep breeds
Source: J Anim Sci Biotechnol. 2023 Nov 6;14:142. doi: 10.1186/s40104-023-00936-y (PMC10626677; doi:10.1186/s40104-023-00936-y)
Supplement: Supplementary file 1 — Additional file 1: Description S1. Contains short description of the breeds and representative pictures of animals used in this study. [file 40104_2023_936_MOESM1_ESM.pdf]

Breed description and phenotype pictures

1. **Istrian Sheep**

Istrian sheep, native to the Istrian peninsula, are believed to have origins in northern Italian breeds like Bergamo sheep. Recognizable by their convex nose, black and white coat, and developed horns, they weigh around 70 kg. As dairy sheep, they produce 135-145 liters of milk during a 6-8 month lactation period.

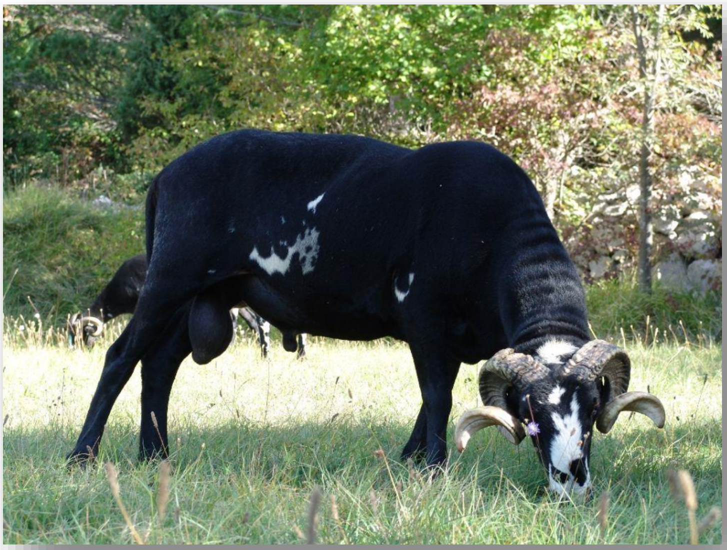

(Source: Croatian Association of Sheep and Goat Breeders)

2. **Cres Island Sheep**

Cres Island Sheep, found on Cres and Lošinj islands, have a history dating to 1332. Crossbreeding with Karakul and Gentile di Puglia breeds took place earlier. They have a smaller build, weighing about 45 kg, with white fleece and hair on legs and belly.

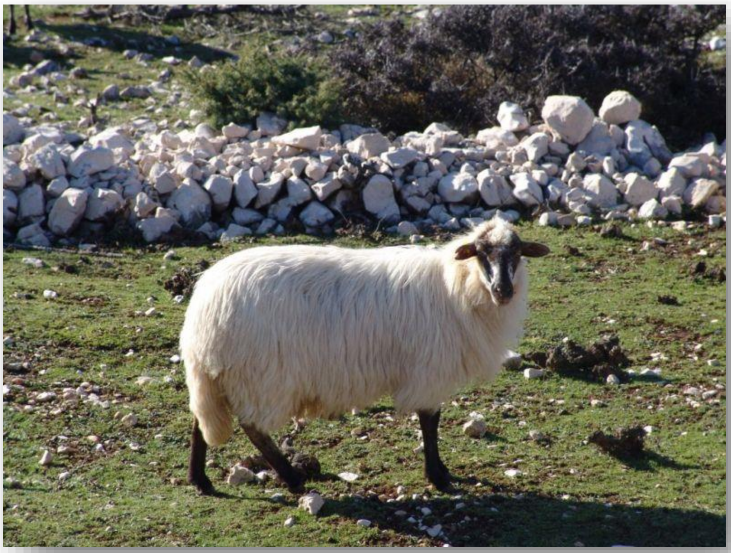

Photo by Ivana Držaić

Breed description and phenotype pictures

3. **Pag Island Sheep**

Pag Island Sheep were created by crossing the native breed with Negretti, Bergamo, and Gentile di Puglia breeds. Genetic improvement began in 1870. Primarily for milk, they're known for Pag cheese and lamb meat. Weighing around 45 kg, they have a distinct fleece and hair combination.

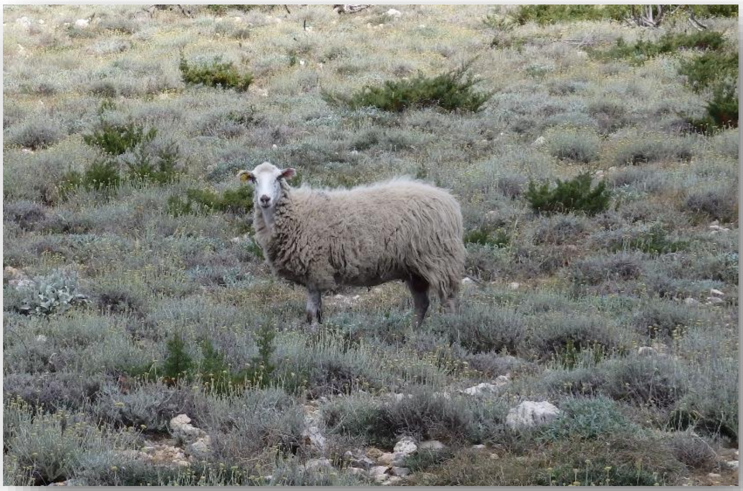

Photo by Ivana Držaić

4. **Krk Island Sheep**

Krk Island Sheep, originating from local sheep and Merino crosses, were first mentioned in the 17th century. Hardy and adaptable, they weigh about 35 kg and feature white or coloured fleece along with hair on their belly and legs.

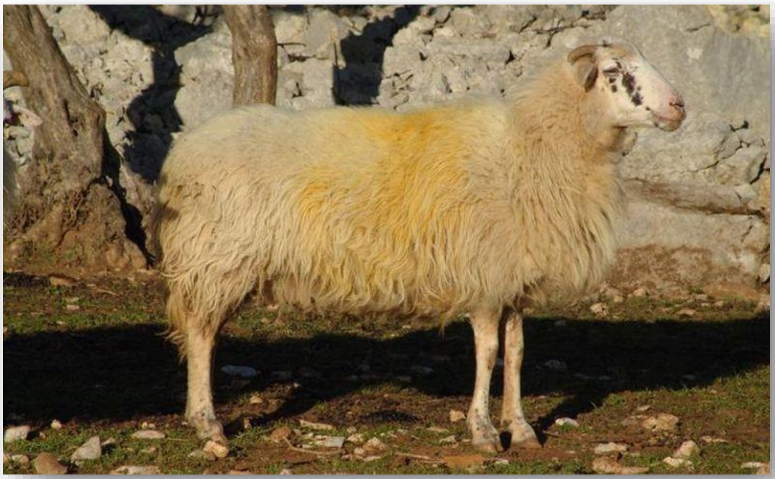

Photo by Ivana Držaić

Breed description and phenotype pictures

5. **Rab Island Sheep**

Exclusively bred on Rab Island, these sheep may carry the merino influence in their lineage. Despite their compact size, they embody robust adaptability, well-suited for the island's terrain. Weighing 30-45 kg, they feature a distinctive semi-closed white fleece, capturing the essence of Rab's pastoral heritage.

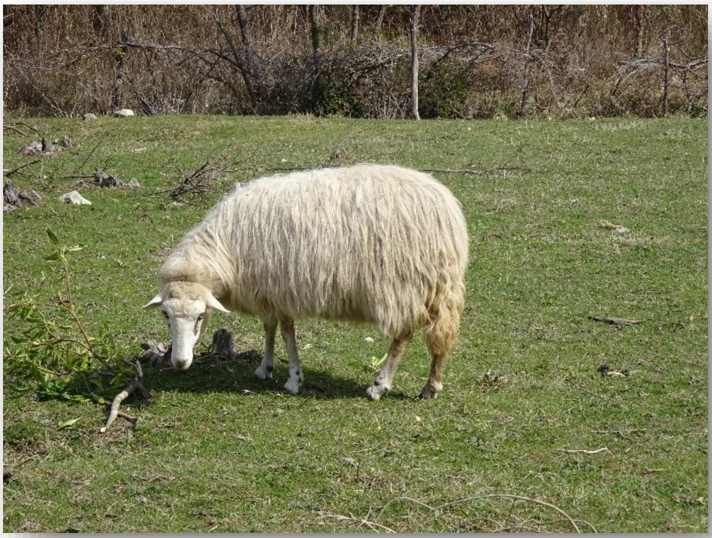

Photo by Ivana Držaić

6. **Lika Pramenka**

Lika Pramenka, hailing from Lika and Gorski Kotar, excels in challenging climates. Crossbreeding introduced foreign influence, maintaining a medium size of about 50 kg. Their distinct appearance includes open white fleece and darker hair on the head and belly.

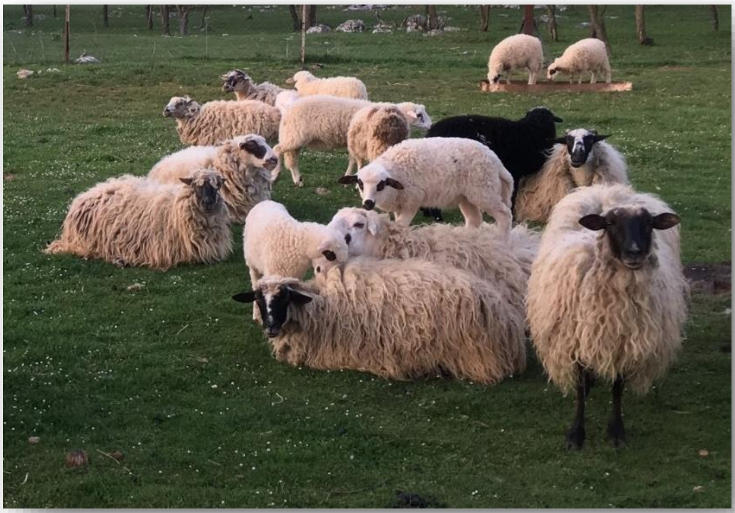

Photo by Vladimir Brajković

Breed description and phenotype pictures

7. **Dalmatian Pramenka**

Dalmatian Pramenka, found across various regions, stands as a sturdy small breed, with a focus on meat production. Their fleece is predominantly white, often accompanied by black-pigmented heads and legs.

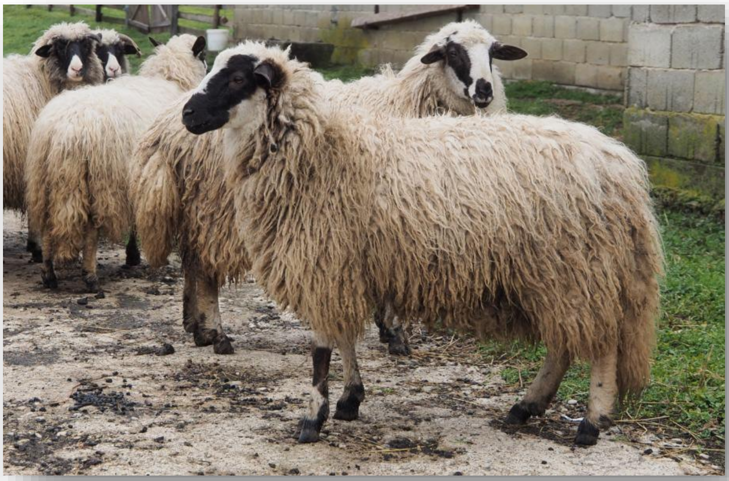

Photo by Ivana Držaić.

8. **Dubrovnik Ruda**

Originating from native Pramenka and Merino crosses, Dubrovnik sheep thrive along the Adriatic coast. Medium-sized at 50 kg, they possess closed to semi-closed white fleece, alongside dense hair on the head, belly, and legs. Despite near extinction, they were successfully revived in the early 2000s.

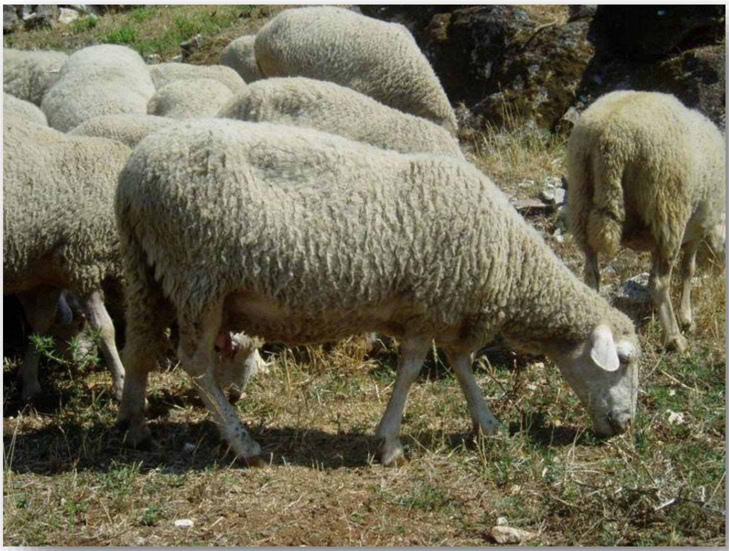

(Source: Croatian Association of Sheep and Goat Breeders)
