# Supplementary figures and images for: Genomic signatures of selection, local adaptation and production type characterisation of East Adriatic sheep breeds
Source: J Anim Sci Biotechnol. 2023 Nov 6;14:142. doi: 10.1186/s40104-023-00936-y (PMC10626677; doi:10.1186/s40104-023-00936-y)

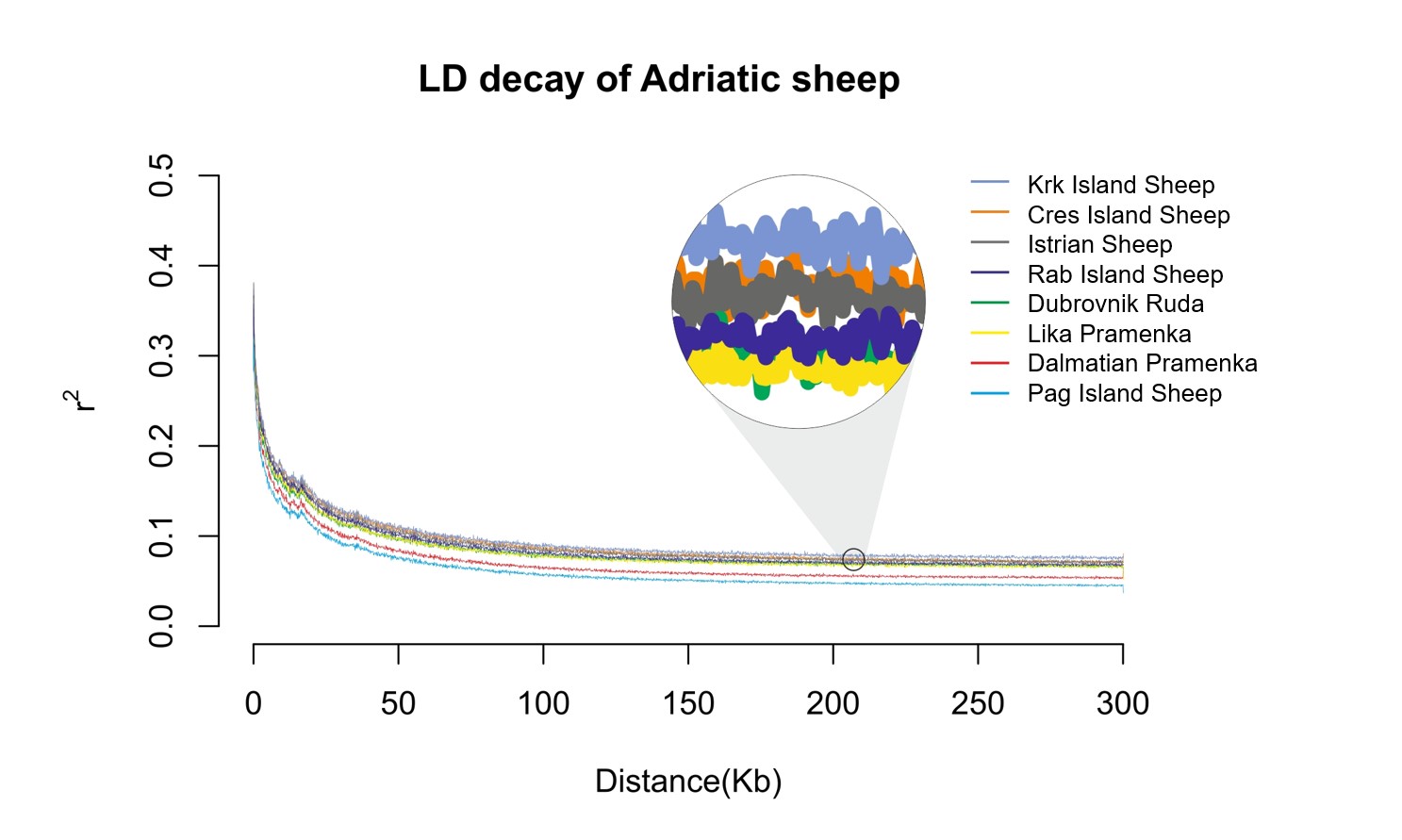

Supplement: Supplementary file 3 — Additional file 3: Fig. S1. LD decay. Contains results from the LD decay analysis. [file 40104_2023_936_MOESM3_ESM.jpg]

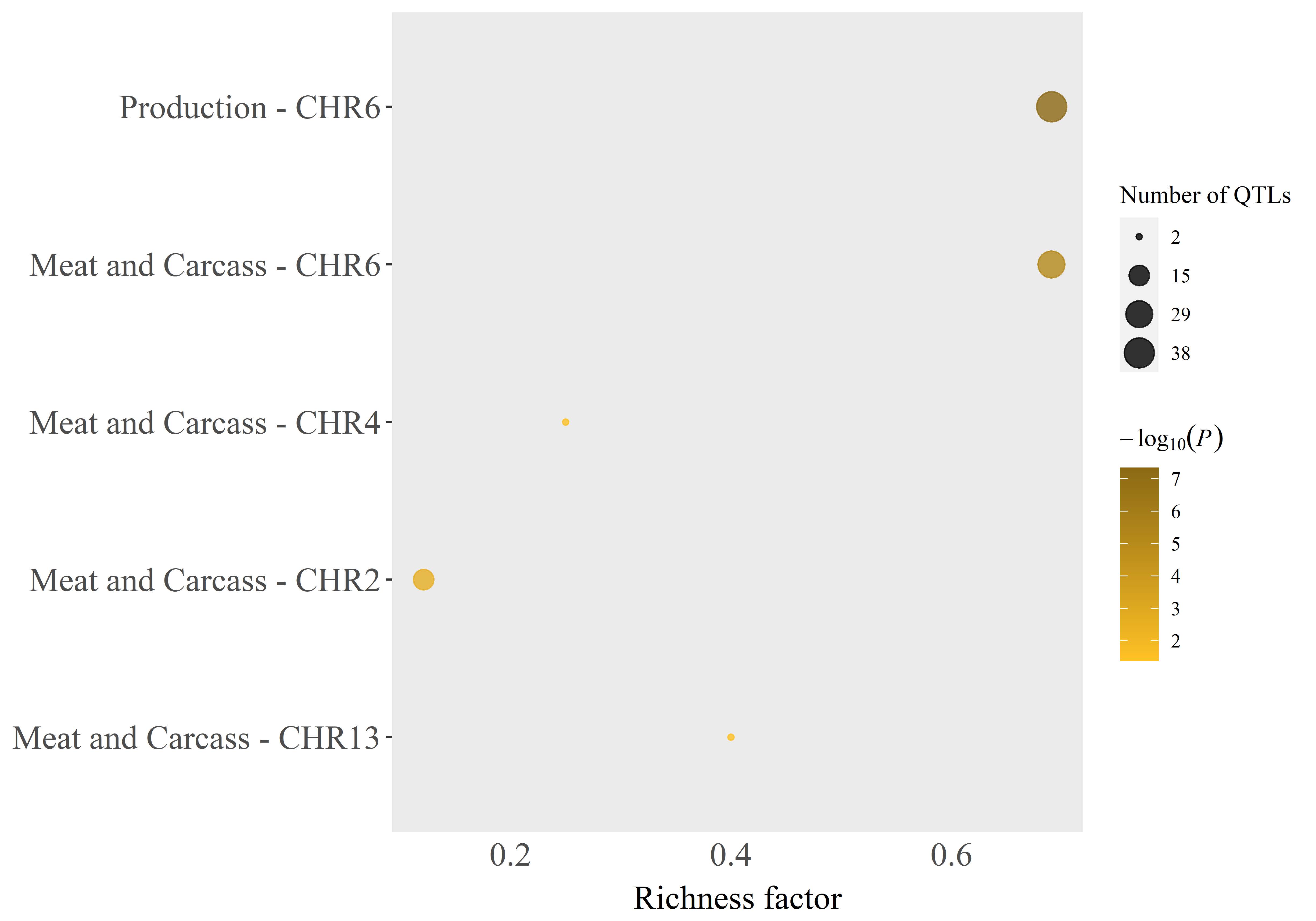

Supplement: Supplementary file 9 — Additional file 9: Fig. S2. Significantly enriched traits per chromosome, as determined by QTL enrichment analysis. The area of the bubbles represents the number of QTLs observed for that class per chromosome, while the colour represents the FDR-adjusted P value as –log10 (P value) (the darker the colour, the smaller the P value). The x-axis shows the richness factor for each QTL, which is the ratio between the number of observed and expected QTLs. [file 40104_2023_936_MOESM9_ESM.jpg]

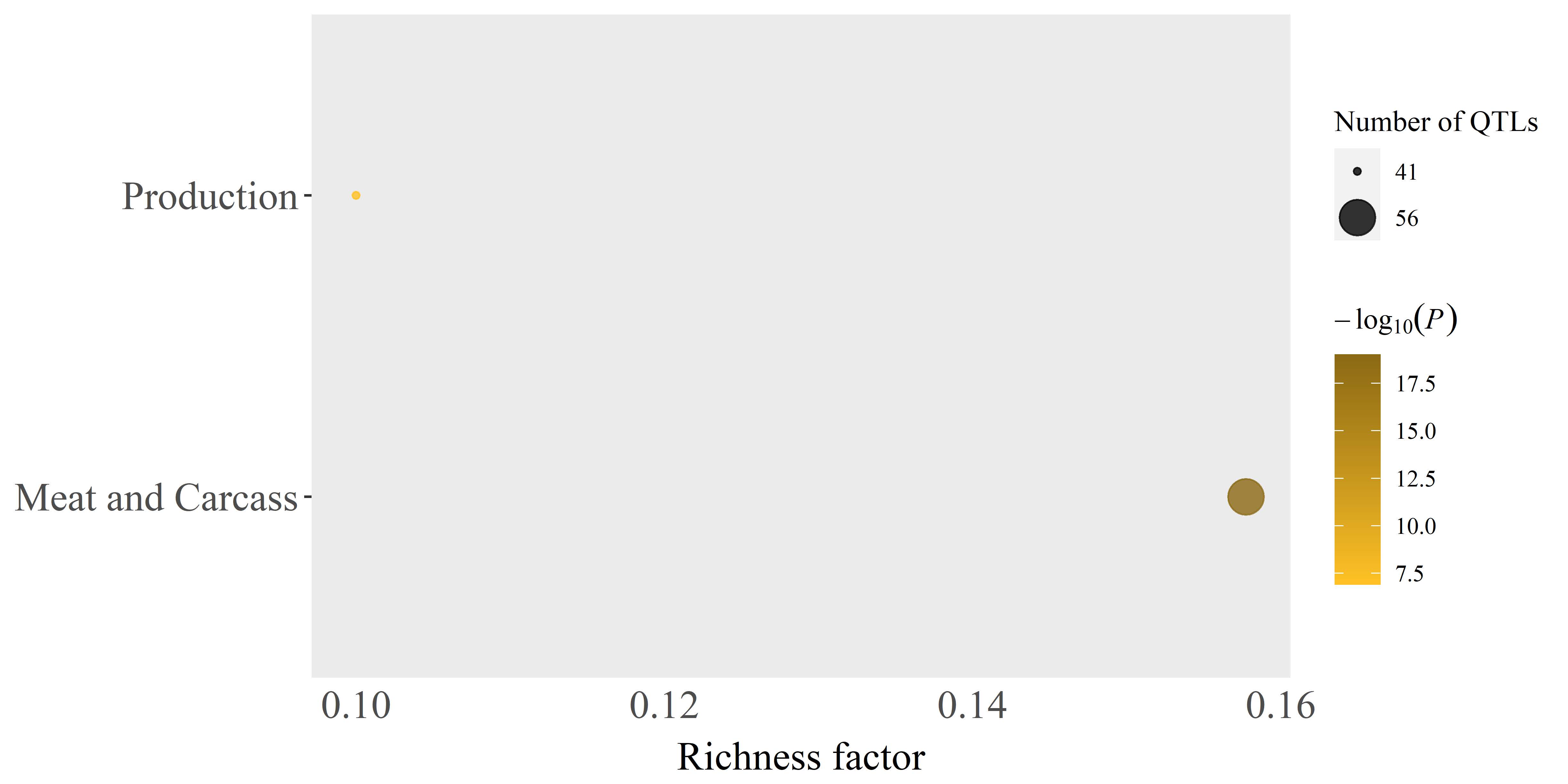

Supplement: Supplementary file 10 — Additional file 10: Fig. S3. Significantly enriched traits per genome, as determined by QTL enrichment analysis. The area of the bubbles represents the number of QTLs observed for that class per genome, while the colour represents the FDR-adjusted P value as –log10 (P value) (the darker the colour, the smaller the P value). The x-axis shows the richness factor for each QTL, which is the ratio between the number of observed and expected QTLs. [file 40104_2023_936_MOESM10_ESM.jpg]
